# The role of alternative pre-mRNA splicing in cancer progression

**DOI:** 10.1186/s12935-023-03094-3

**Published:** 2023-10-24

**Authors:** Sunkyung Choi, Namjoon Cho, Eun-Mi Kim, Kee K. Kim

**Affiliations:** 1 Department of Biochemistry, College of Natural Sciences, Chungnam National University, Daejeon, 34134 Republic of Korea; 2 Department of Predictive Toxicology, Korea Institute of Toxicology, Daejeon, 34114 Republic of Korea

**Keywords:** Alternative pre-mRNA splicing, Cancer, Cell proliferation, Signaling pathway, Splicing factor

## Abstract

Alternative pre-mRNA splicing is a critical mechanism that generates multiple mRNA from a single gene, thereby increasing the diversity of the proteome. Recent research has highlighted the significance of specific splicing isoforms in cellular processes, particularly in regulating cell numbers. In this review, we examine the current understanding of the role of alternative splicing in controlling cancer cell growth and discuss specific splicing factors and isoforms and their molecular mechanisms in cancer progression. These isoforms have been found to intricately control signaling pathways crucial for cell cycle progression, proliferation, and apoptosis. Furthermore, studies have elucidated the characteristics and functional importance of splicing factors that influence cell numbers. Abnormal expression of oncogenic splicing isoforms and splicing factors, as well as disruptions in splicing caused by genetic mutations, have been implicated in the development and progression of tumors. Collectively, these findings provide valuable insights into the complex interplay between alternative splicing and cell proliferation, thereby suggesting the potential of alternative splicing as a therapeutic target for cancer.

## Introduction

Most human pre-mRNAs contain two regions: one is the exon, which codes for the mRNA and produces the specific protein, and the other is the intron, which does not code for the protein sequence and which is removed from the pre-mRNA by a sophisticated biological process called RNA splicing. Although the intronic sequences are not pivotal for protein translation, many eukaryotic genes in yeasts to vertebrates possess them. Interestingly, the higher the evolutionary level of the species, the longer and more complicated the intronic sequence [1, 2].

The complexity of intron sequences promotes alternative splicing, in which exons are selectively included in the mRNA or the introns are not excluded. If alternative splicing events alter the combinations of exons that possess protein-coding sequences (CDS), the resulting alteration in protein products may affect their subcellular localization, secretory activity, stability, enzymatic activity, post-translational modification (PTM), or protein interactions [3–5]. Even if alternative splicing changes only the untranslated region (UTR) in the mRNA, the translation efficiency or mRNA stability could change between splicing-generated mRNA variants [6, 7]. Since 95% of multi-exon human genes undergo alternative splicing, systemic changes in alternative splicing can regulate most cellular processes including cell growth and survival [8]. Although a wide range of alternative splicing events may occur by chance because of misprocessed RNA splicing, many alternative splicing events are highly conserved among vertebrates, and they play a crucial role in determining cell fates [9]. These events are mostly finely regulated by the expression and activation of splicing factors according to tissue type, developmental stage, and signal transduction [10, 11]. However, genetic mutations in the splicing factor or *cis*-regulatory RNA element, as well as abnormal expression of the splicing factor, can induce aberrant alternative splicing, which can promote the expression of undesired protein isoforms instead of the appropriate protein isoforms, leading to the progression of human diseases, including cancer [12].

Comprehensive analyses of alternative splicing from patients with cancer have identified the global alteration of splicing pools in cancer tissues [13]. Additionally, recent studies have demonstrated that alteration of alternative splicing in specific genes is potentially associated with oncogenic properties including cell growth and survival [14]. Appropriate cell proliferation monitored through checkpoints is essential for growth, regeneration, and maintenance of tissue functions. Cells can arrest the cell cycle when they sense DNA damage or undergo apoptosis because of excessive DNA damage with unsuccessful DNA repairs [15, 16]. They can exit the cell cycle by differentiating into specific cell types or entering a quiescent stage. However, cancer cells display uncontrolled cell cycle progression and resistance to cell death [17, 18]. These cancer cells exhibit transcriptomic abnormalities that promote cancer cell transformation and proliferation. Aberrant alternative splicing may play a critical role in these transcriptome abnormalities by affecting expression of specific cancer-associated isoforms.

In this review, we describe the current understanding of the role of alternative splicing in controlling cell division and survival by discussing specific splicing factors and isoforms and their molecular mechanisms for regulating cell proliferation. Finally, we provide a novel insight that the proteome diversity generated by alternative splicing is a key to resolving the complexity of cell proliferation mechanisms and to understanding the detailed mechanism underlying human diseases related to cell proliferation.

## Regulatory mechanism of alternative splicing

**Fig. 1:**
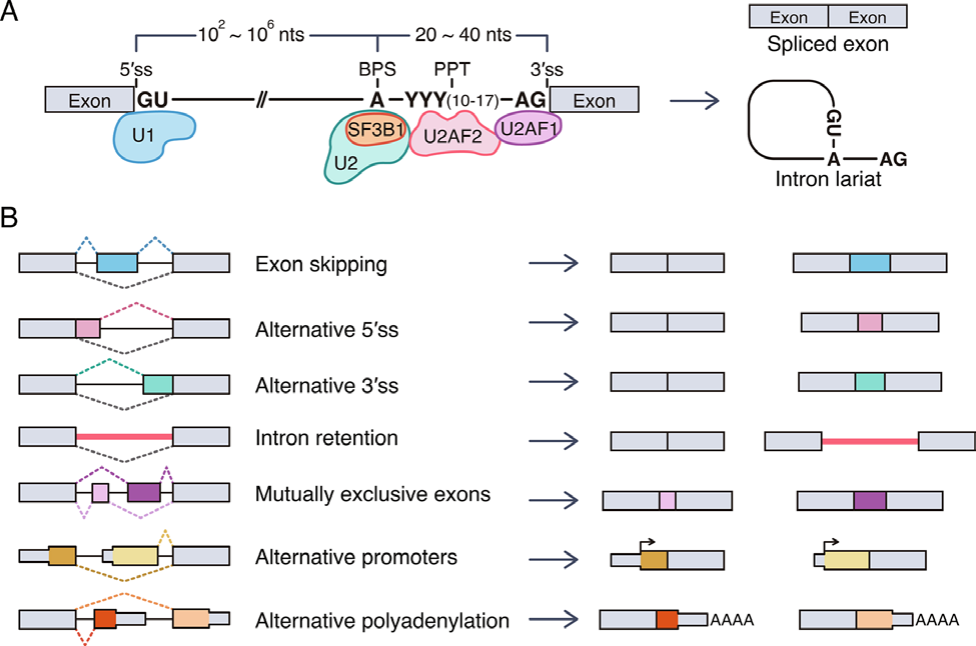
Overview of splicing mechanisms and alternative splicing types. **(A)** Simplified schematic illustration of pre-mRNA splicing. Evolutionarily conserved 5′ splice sites (5′ss; GU) and 3′ splice sites (3′ss; AG) are located at the 5′ and 3′ ends of introns, respectively. Within the upstream intron of the 3′ss, a branchpoint sequence (BPS) and a polypyrimidine tract (PPT) are present. The 5′ss is recognized by the U1 snRNP, the PPT by the U2AF2, and the 3′ss by the U2AF1. The U2 snRNP recognizes the BPS. It then undergoes two successive transesterification steps to generate splicing products (spliced exon and lariat intron). **(B)** Major types of alternative splicing events. Cells generate different mRNA transcripts by exon skipping of alternative exons, the selection of alternative 5 or 3′ss, retention of introns, selection of the mutually exclusive exons, and selection of alternative promoters or polyadenylation. Exons are represented by colored boxes, introns by horizontal lines, and distinct alternative splicing events by dotted lines

Splicing occurs through a sequential catalytic process involving the spliceosome complexes U1, U2, U4, U5, and U6, in the canonical splicing process [19]. The spliceosome complexes comprise small nuclear ribonucleoproteins (snRNPs) consisting of small nuclear RNA (snRNA) and numerous proteins [20]. During the transcription of multi-exon genes, splicing occurs simultaneously to remove introns from the transcripts via recruitment of spliceosome complexes. The primary criterion for defining introns and exons within the pre-mRNA is the presence of intronic consensus sequences located at the 5′ and 3′ splice sites (ss). These sequences are recognized by the snRNA of the spliceosome via base-pairing with the target pre-mRNA [21, 22] (Fig. 1A). The U1 snRNP binds to the GU motif at the intron boundary of the 5′ss. SF1 and U2AF2 bind to the branch point site (BPS) and polypyrimidine tract (PPT), respectively, and U2AF1 binds to the AG motif at the intron boundary of the 3′ss. Subsequently, SF1 protein is released; U2 snRNP binds to the BPS, and it is stabilized by SF3B1. Next, the U4/U6•U5 tri-snRNP complex is recruited, following which U1 and U4 snRNPs are released to form a catalytically activated spliceosome. Finally, the RNA undergoes conformational rearrangement and is catalyzed in two transesterification steps: one involves 5′ss cleavage to form a lariat intron and the other involves catalysis of exon ligation. Consequently, mRNA is generated, and snRNPs are recycled for additional splicing processes.

Although splicing occurs according to splice site sequences, several human introns frequently have weak intronic consensus sequences [2, 23]. Moreover, numerous splicing factors are expressed, they bind to intronic or exonic splicing regulatory sequences, and these *trans*-acting splicing factors contribute to or interfere with the recruitment of spliceosome to the splice site, depending on the specific site to which they bind [24]. Consequently, splicing factors affect the splice site selection of spliceosome, particularly showing greater effects on splice site selection in introns containing weak consensus sequences [25]. Therefore, although the transcripts are expressed from one gene, depending on the expression and activation of splicing factors, mRNA can be expressed as different combinations of exons, producing alternative splicing variants. In particular, in higher mammals, alternative splicing plays a pivotal role in providing complexity to cellular system regulation by diversifying the transcriptome and fine-tuning gene functions [23].

Most alternative splicing events can be classified into seven types according to the pattern of splice site changes (Fig. 1B). Exon skipping is the most predominant event of alternative splicing in humans. Specific exons that are included or excluded in the mRNA are called cassette exons. Alternative 5′ss or 3′ss change the length of the exon. Some introns can be retained in the mRNA. Intron sequences mainly encode nonfunctional protein sequences and involve the termination codon, potentially leading to a reduction of protein function. Mutually exclusive exons represent an alternative splicing pattern in which only one exon is included between consecutive alternative exons. Lastly, alternative promoters or alternative polyadenylation generate different splicing variants.

Comprehensive bioinformatics analyses have revealed that alternative splicing globally and dynamically occurs in most human genes as various patterns of exon selection [9]. To explore the detailed function of genes for further understanding of complex cellular mechanisms, such as cell proliferation and survival, the study of alternative splicing patterns of transcripts and their differential functions of protein isoforms is indispensable, especially in understanding human disease.

## Alternative splicing of pathway components related to cancer cell growth

### Cell cycle pathway

**Fig. 2:**
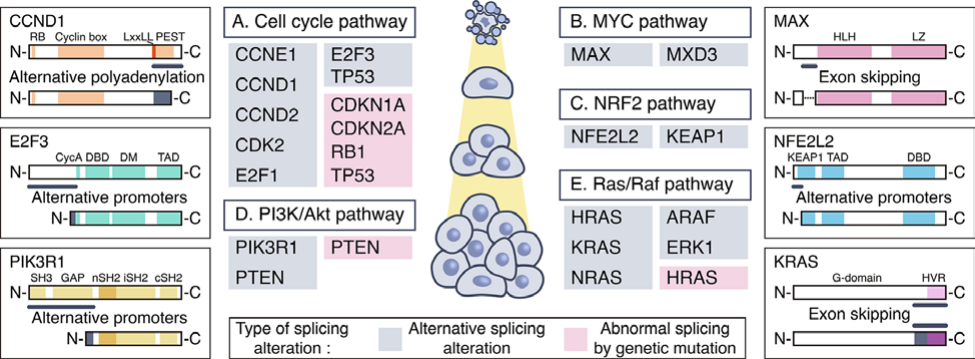
Key signaling pathways that affect cancer cell growth and alternative splicing events that regulate these pathways. Alternative splicing is involved in the regulation of significant signaling transduction pathways that affect cancer cell growth. The five major signal transduction pathways that affect cancer cell growth include the cell cycle, MYC, NRF2, PI3K/Akt, and Ras/Raf pathways. Through alternative splicing, genes within these pathways can generate diverse protein isoforms with distinct functions and expression levels. Through alternative splicing, these genes can dynamically modulate the signaling landscape, contributing to the regulation of cancer cell growth. Genes regulating these pathways through alternative splicing are shown in gray boxes, and genes with abnormal splicing due to genetic mutations are shown in pink boxes. Representative alternative splicing events among these genes are depicted. The main domains of these genes are marked with colored boxes, and the domain regions changed according to alternative splicing and the type of alternative splicing are indicated

The mitotic cell cycle is a process in which duplicated chromosomes are separated into two cells to obtain two genetically identical daughter cells (Fig. 2A). Chromosomes are replicated through two stages, interphase and M phase. The cell cycle is controlled by complex mechanisms such as cell cycle checkpoints that monitor the appropriate cell size, complete chromosome replication, and correct cell segregation for timely and accurate cell division [26]. The main mechanism of action of the cell cycle checkpoint is to regulate the activity of cyclin-dependent kinases (CDKs), a family of serine/threonine protein kinases. Cyclin/CDK complexes are formed and activated at various stages of the cell cycle, and they control cell cycle progression through phosphorylation of downstream targets such as the tumor suppressor retinoblastoma (Rb) [27].

In keratinocytes, increased eIF2α phosphorylation by UVB enhanced translation of cyclin dependent kinase inhibitor 1 A (*CDKN1A*, also called *p21*) transcript variant 4 through a mechanism mediated in part by upstream open reading frames (ORFs) situated in the 5′-leader of *CDKN1A* mRNA [28]. eIF2α phosphorylation protects cells from stress, such as UVB irradiation, by regulating cell cycle control and determining cell fate through a specific splicing variant of *CDKN1A* that promotes G1 arrest and subsequent DNA repair. In addition, a mutation that destroys the splice site of *CDKN1A* has been found to induce the skipping event of exon 2 and, interestingly, cause transcript fusion between *CDKN1A* and *RAB44*, which are different genes [29].

Cyclin dependent kinase inhibitor 2 A (*CDKN2A*), located on human chromosome 9, encodes the p16^INK4A^ and p14^ARF^ proteins. A splice site mutation in *CDKN2A*, found in a family with melanomas, neurofibromas, and multiple dysplastic nevi, was reported to cause the skipping of exon 2, which encodes more than 50% of the p16^INK4A^ and p14^ARF^ proteins [30]. In addition, splice site variation of *CDKN2A* was found by sequencing of 167 melanoma-prone families [31]. The AGgt-to-AGtt and AGgt-to-ATgt mutations in *CDKN2A* disrupted the normal splicing of exon 2. The AGgt-to-CGgt mutation of exon 1 disrupted splicing and affected the function of p16^INK4A^, confirming that the splice site mutation plays an important role in melanoma susceptibility.

Cyclin E1 encoded by *CCNE1* controls the G1/S transition. In the *Ccne1* splicing variant IN3 (ORF shifted from exon 2 to exon 3 due to alternative 5′ss selection), exon 4 skipping and exon 5 skipping are associated with retarded proliferation in murine hepatocellular carcinoma [32]. Isoforms with partial loss of exons 3 and 8 and complete loss of exons 4, 5, 6, and 7 are localized in the cytoplasm due to the lack of a nuclear localization signal. This Ccne1 isoform is predominantly expressed in G0 in hepatocytes and forms inactive complexes to sequester CDK2 in the cytoplasm, delaying cell cycle re-entry of hepatocytes after G1 arrest.

Human cyclin D1 (*CCND1*) splicing occurs at the exon 4/intron 4 boundary. When splicing occurs at this site, the cyclin D1a protein is produced; when splicing fails, the cyclin D1b protein, which contains a part of intron 4 instead of exon 5 and terminates prematurely, is produced. Cyclin D1b splicing is increased by G/A870 polymorphism and SRC-associated in mitosis of 68 kDa (SAM68) [33, 34]. Cyclin D1b is upregulated in various carcinomas, including prostate cancer, and exhibits a higher carcinogenic potential than cyclin D1a because of its stronger nuclear localization [35–37]. The cyclin D2 (*Ccnd2*) splice variant found in the mouse heart contains exon 1 and the longer exon 2, which generate 20 novel residues in the C-terminal [38]. This isoform aggregates in the endoplasmic reticulum (ER), Golgi, and lysosomal compartments and functions as a negative cell cycle regulator by aggregating and sequestering several cell cycle proteins. The 17 kDa truncated cyclin D2 isoform localizes to the cytoplasm and interacts with CDK4; however, the complex is unable to phosphorylate the target pRb [39].

The long isoform of CDK2 with 48 residues inserted owing to partial insertion of intron 5 has approximately half the specific activity compared to the normal form of CDK2 when forming a complex with cyclin A [40]. Poly(C)-binding proteins (PCBPs) bind to C-rich PPT in intron 4 and promote inclusion of *CDK2* exon 5 [41]. Exon 5 exclusion of *CDK2* dramatically reduces CDK2 protein expression, affecting cell cycle kinetics.

E2F transcription factor 1 (E2F1) is a transcription factor that regulates the S phase transition from G0/G1. Intron 5 retention and exon 6 skipping variants were identified in the central nervous system of rats, and these were terminated early, resulting in C-terminal truncation [42]. E2F transcription factor 3 (E2F3) produces isoforms called E2F3a and E2F3b owing to an alternative promoter [43, 44]. E2F3a is tightly regulated by cell growth and is expressed only at the G1/S boundary, whereas E2F3b is detected throughout the cell cycle and expressed in both quiescent and proliferating cells. Additionally, in quiescent cells, the E2F3b protein binds to Rb, resulting in a predominant E2F-Rb complex [45].

In RB1, the G1/S checkpoint regulator, 27% of coding mutations disrupted splicing [46]. Of the *RB1* exon splicing mutations, 58% were blocked primarily at the A complex converting to the B complex, and 33% at the B complex. Mutation-induced *RB1* exon loss or intronic sequence exonization induced premature termination, resulting in the restriction of RB1 expression [47, 48]. Inhibition of RB1 expression induced a high expression of p16^INK4A^. In addition, the *RB1* splicing mutation is associated with prognosis and low penetrance in patients with non-small cell lung cancer (NSCLC) or retinoblastoma [47, 49].

The tumor suppressor p53 induces cell cycle arrest at multiple stages, including G/S and G2/M checkpoints, in situations such as DNA damage. The use of an alternative 3′ss located in tumor protein P53 (*TP53*) intron 6 results in the p53Ψ isoform lacking major parts of the DNA-binding domain, nuclear localization sequence, and tetramerization domain [50]. The p53Ψ isoform is not capable of DNA binding and transactivation, but it induces mesenchymal-like characteristics and improves motility and invasion of normal and malignant cells. *TP53*, a gene encoding the tumor suppressor protein p53, called “guardian of the genome”, has been reported to have splice site mutations in patients with various cancers, including colorectal cancer [51, 52]. Exon 4–intron 4 junctions mutations identified in pediatric adrenocortical tumors induce erroneous splicing, resulting in protein instability, altered intracellular localization, and loss of function [53]. Exon 6-truncating mutants, similar to the p53Ψ isoform, lack transcriptional activities and the ability to respond to DNA damage, as well as pro-tumorigenic functions that promote cancer cell proliferation, survival, and metastasis [54].

### MYC pathway

The MYC oncogene family consists of three members: *MYC*, *MYCN*, and *MYCL*, which encode c-Myc, N-Myc, and L-Myc, respectively (Fig. 2B). Myc is a basic helix-loop-helix and leucine zipper (bHLH-LZ) transcription factor that regulates approximately 15% of the total transcriptome [55, 56]. Thus, Myc proteins mediate various biological processes including cell growth and proliferation, apoptosis, differentiation, cell cycle, and metabolism [57]. Myc promotes transcription by forming a heterodimer with Myc-associated factor X (MAX) through the bHLH-LZ domain required for DNA-protein interaction. Unlike Myc, which forms a heterodimer only with MAX, MAX forms a homodimer or binds to the MXD, MGA, and MNT proteins [58]. This process induces an antagonistic effect on the Myc family by sequestering MAX from Myc.

MAX with exon 2 inclusion was considerably more effective at binding homodimeric DNA than exon 2 skipping of MAX [59]. In addition, this isoform induced a decrease in Myc expression, slowed growth, and accelerated apoptosis during growth factor deprivation. In another MAX isoform, the basic region, helix 1, and the loop of the helix-loop-helix region were deleted [60]. Since this MAX isoform cannot bind to E-box Myc site DNA because the basic region does not exist, it appears to function as a dominant negative regulator. The MAX protein with a C-terminus truncated by intron 4 retention was shown to retain its ability to bind to the CACGTG motif in complex with c-Myc; however, it was located in the cytoplasm [61]. In addition to the wild type of MAX, hypoxia induced intron 4 retention and alternative splicing including cassette exon located within intron 4 [62]. The isoforms produced by intron 4 retention are highly destabilized by 36 isoform-specific amino acids, which destabilize heterologous proteins. The variant containing the cassette exon located within intron 4 is degraded by nonsense-mediated mRNA decay (NMD), and both isoforms play a role in downregulating the wild-type MAX isoform.

MAX dimerization protein 3 (*MXD3*) has a variant that uses exon 6 as the last exon and a variant that uses exon 7 instead of exon 6 as the last exon [63]. These two splice variants are most likely a result of alternative polyadenylation. According to an analysis of The Cancer Genome Atlas (TCGA) data, exon 7 mRNA was expressed at higher levels in normal cells than in cancer cells, whereas exon 6 mRNA was expressed at higher levels in cancer cells. The exon 7 inclusion isoform containing a considerably longer 3′UTR has a greater reduction in protein expression compared to the exon 6 inclusion isoform. In addition, the exon 7 inclusion isoform undergoes phosphorylation, and it is localized throughout the nucleus; in contrast, the exon 6 inclusion isoform does not appear to be phosphorylated, and is mainly confined to the nuclear foci.

### NRF2 pathway

The NRF2 pathway regulates transcriptional responses of genes important for oxidative and electrophilic stress responses (Fig. 2C) [64–66]. Kelch-like ECH-associated protein 1 (Keap1), a substrate adaptor for a cullin-3 (Cul3)-based ubiquitin ligase, inhibits the transcriptional activity of nuclear factor-erythroid factor 2-related factor 2 (Nrf2) by promoting ubiquitination and proteasomal degradation of the transcription factor Nrf2 under basal conditions [67–70]. Thiols of Keap1 cysteines are modified by oxidants, electrophiles, and Nrf2 activators, and Nrf2 is dissociated from Keap1 [65, 71]. The dissociated Nrf2 translocates to the nucleus without ubiquitination and proteasome degradation, forming a heterodimer with small musculoaponeurotic fibrosarcoma oncogene homologue (sMAF) [72, 73]. The Nrf2/sMaf heterodimer binds to the antioxidant response element (ARE) and induces the transcription of a battery of antioxidant and detoxification genes [74–76].

Exon 2 or exon 2 + 3 skipping of NFE2 like BZIP transcription factor 2 (*NFE2L2*), the gene coding for Nrf2, was found in some NSCLCs and patients with head and neck squamous carcinoma (HNSC) [77]. These abnormal transcript variants lack the Keap1 interaction domain, resulting in loss of interaction with Keap1. Thus, Nrf2 is stabilized, resulting in an Nrf2 transcriptional response and Nrf2 pathway dependence. *NFE2L2* can be spliced using a second alternative promoter (P2) that exists downstream. Because these P2 transcripts start translation using a different AUG than those that use the upstream promoter (P1), they miss the part of exon 1 that encodes the 16 amino acids and thus have a shorter N-terminus. The ΔN-Nrf2, a protein of the NFE2L2-P2 transcript, is more stable and abundant in cells in the absence of stress owing to its impaired binding to Keap1 [78]. Tert-butylhydroquinone (tBHQ) induces electrophilic stress, and the full-length protein isoform of the NFE2L1-P1 transcript was strongly detected in the nucleus, whereas ΔN-Nrf2 translocation from the cytoplasm to the nucleus was not observed. Further studies are needed to determine the function of ΔN-Nrf2 in the cytoplasm, in addition to its transcriptional activity, and whether this function is related to carcinogenesis.

*Keap1* splice variants were found in human highly-metastatic hepatoma (MHCC97H) cells and other cell lines. This *Keap1* splice variant (Keap1ΔC) lacks exons 4 and 5, resulting in the loss of 180 amino acid residues [79]. Since the missing amino acid residues are located in most of the double glycine-repeat (DGR) domain and the adjacent C-terminal region essential for interaction with Nrf2, these Keap1 isoforms retain no or little ability to inhibit Nrf2. Therefore, the isoform in which exons 4 and 5 are skipped acts as a dominant-negative competitor of intact Keap1 because of its antagonist effect on Keap1-mediated turnover of Nrf2.

### PI3K/Akt pathway

The PI3K/Akt pathway is involved in several cellular physiological processes such as cell cycle, growth, proliferation, survival, and autophagy (Fig. 2D). PI3K activation is stimulated by various oncogenes and growth factor receptors, resulting in the conversion of phosphatidylinositol (3,4)-bisphosphate (PIP2) to phosphatidylinositol (3,4,5)-trisphosphate (PIP3) [80]. PIP3 serves as a plasma membrane docking site for the recruitment and activation of several pleckstrin homology (PH) domain-containing proteins such as AKT, SGK, and PDK1 [81]. Phosphorylation of Akt Thr308 by PDK1 at the membrane leads to partial activation of Akt, and Ser473 phosphorylation by a complex involving mTOR/Rictor (TORC2) results in full activation of this enzyme. Activated Akt phosphorylates downstream effectors, including GSK3β, FoxO, MDM2, and BAD, in the cytoplasm and nucleus, triggering various biological processes such as cell cycle, apoptosis, and glucose metabolism. Thr308 and Ser473 of Akt are dephosphorylated by PP2A and PHLPP1/2, respectively, and PTEN dephosphorylates PIP3 to inhibit Akt activity [82–84]. Akt causes TSC2 phosphorylation and inactivation and induces RHEB to activate the mTOR/Raptor (TORC1) complex, which affects mRNA translation, nucleotide and lipid synthesis, cell growth, and autophagy.

PI3K is a heterodimeric enzyme composed of a p110 catalytic subunit and a regulatory subunit. The phosphoinositide-3-kinase regulatory subunit 1 (*PIK3R1*) encodes the PI3K regulatory subunits p85α, p55α, and p50α, with the same C-terminal but different N-terminals as a result of alternative splicing. The p85α protein with the longest N-terminal region can form a homodimer through SH3 domain-PR1 (proline-rich motif 1) and BH-BH domain interactions [85–87]. Additionally, p85α binds to PTEN through the N-terminal SH3-BH region, which is absent in p55α and p50α, and enhances enzymatic activity. Through this process, p85α plays a role in maintaining the balance of PI3K signaling by regulating not only p110-PI3K but also PTEN-PI3K. Stat3 directly binds to the p55α and p50α promoters in vivo to induce their expression, and the overexpression of p55α or p50α reduced the level of activated Akt [88]. *PIK3R1* splice acceptor and donor sites mutations in exon 11 induce exon 11 exclusion, resulting in a protein lacking a portion (Δ434_475) of the iSH2 domain [89, 90]. The protein thus produced has been shown to interfere with the regulation of p110δ, resulting in increased p110δ signaling and triggering activated phosphoinositide 3-kinase δ syndrome (APDS) type 2, which causes primary antibody deficiency, senescent T cells, developmental abnormalities within the T lymphocyte compartment, and immune dysregulation [91–95].

The isoform generated by partial insertion of intron 8 of PTEN lacks part of the C2 domain, C-tail, and PDZ-binding domains [96]. Consequently, it induces reduced cell migration, adhesion, and enhanced apoptosis, which, similar to the full-length isoform, acts as a tumor suppressor. Additionally, studies have reported variants containing segments (3a, 3b, 3c; 5a, 5b, 5c) of different sizes of intron 3 or intron 5 and variants in which a part of exon 5 or all of exon 6 were excluded [97, 98]. The *PTEN* variant was regulated by p53 and differentially expressed in heritable breast cancer syndrome, sporadic breast cancers, and Cowden syndrome compared with the normal tissues. The PTEN 5a isoform functions similar to full-length PTEN by reducing cyclin D1 promoter activity and Akt phosphorylation; in contrast, PTEN 5b and 5c appear to exert the opposite function by decreasing cyclin D1 promoter activity. Mutations in the splice junction or intron region of the *PTEN* gene have been identified in patients with Cowden syndrome and Bannayan Zonana syndrome, an autosomal dominant genetic disorder characterized by hamartomas [99, 100]. These mutations caused either out-of-frame skipping of an entire exon or activation of cryptic splice sites, resulting in partial intronic sequence inclusion, premature transcript termination, or polyadenylation within exon 8. Samples with splicing changes due to these mutations exhibited lower PTEN protein expression and higher Akt phosphorylation compared with samples without splicing changes even with mutations; however, p-ERK1/2 did not increase. These findings suggest that PTEN variants may contribute to the pathogenesis of various diseases and may serve as diagnostic markers.

### Ras/Raf pathway

Consisting of the Ras-Raf-MEK-ERK signaling cascade, the mitogen-activated protein kinase (MAPK) pathway is an essential cellular network for inter- and intra-cell communication that transmits, amplifies, and integrates signals from various stimuli, such as growth factors, hormones, tumor-promoting substances, and differentiation factors (Fig. 2E). This pathway activates transcription factors and regulates genes that control cell proliferation, differentiation, angiogenesis, inflammation, development, apoptosis, integrin signaling, and migration. Ras small GTPase, the first component of MAPK signaling, contains three genes, *HRAS*, *NRAS*, and *KRAS*, that encode four RAS proteins (HRas, NRas, KRas4A, and KRas4B) [101]. The Raf family comprises three genes (*ARAF*, *BRAF*, and *RAF1*), and the MEK family, five genes (*MEK1*, *MEK2*, *MEK3*, *MEK4*, and *MEK5*). When the ligand binds to the tyrosine kinase receptor, the guanine exchange factor is recruited and activated, leading to the exchange of GDP with GTP in Ras. Activated Ras induces Raf activation to form Ras homo- or heterodimer. Active RAF dimers recruit MEK and subsequently activate ERK. Activated ERK1/2 alters gene expression in cells by phosphorylating multiple substrates and regulating various transcription factors. Conversely, when Ras becomes inactive GDP-bound state by GTPase activating protein, downstream signaling is turned off.

*HRAS* exon 2 has a relatively weak 3′ss; therefore, inclusion and exclusion are regulated by the balance of positive and negative splicing regulatory factors such as SRSF2 and hnRNPF/H [102]. Therefore, exon 2 mutation identified in attenuated patients with Costello syndrome have been shown to simultaneously interfere with exonic splicing enhancer function and exonic splicing silencer generation, causing exon 2 skipping. These splicing abnormality abrogated the production of HRAS protein and inhibited cancer cell proliferation. Since HRAS exon 2 inclusion may also affect the onset of Costello syndrome and carcinogenic potential, splice switching oligonucleotides that induce exon 2 exclusion can serve as a suitable strategy for therapy.

*KRAS* generates K-Ras4A and K-Ras4B according to the use of the alternative fourth exon. Exon 4 encodes HVRs, a membrane binding and targeting motif, and K-Ras4A and 4B are differentially expressed in mouse development and adult tissues [103, 104]. K-Ras4A is palmitoylated, whereas K-Ras4B lacks a palmitoylation site [103–105]. Thus, in addition to the CAAX motif, K-Ras4A has a dual membrane-targeting motif, as a site of palmitoylation and bipartite polybasic region exists at its C-terminus. Therefore, K-Ras4A plays an important role in K-Ras-induced tumors, and the mechanism by which K-Ras4A accesses the plasma membrane differs from that of K-Ras4B [106]. Additionally, unlike K-Ras4B, K-Ras4A does not bind to the cytosolic chaperone δ-subunit of cGMP phosphodiesterase type 6 (PDE6δ); therefore, a significant difference has been observed in their subcellular trafficking. K-Ras4A has been shown to respond to hypoxia and K-Ras4B to ER stress; KRAS4A splicing is controlled by the DCAF15/RBM39 pathway [107]. Cells with a low KRAS4A/KRAS4B ratio have shown higher sensitivity to cancer treatment drugs and an association with high KRAS signaling and a poor patient outcome, suggesting that they could serve as biomarkers of sensitivity to existing cancer treatments [107, 108].

Five splicing isoforms have been reported for NRAS: isoform 1; isoform 2 containing exon 3b; isoform 3 lacking exon 3; and isoform 4 lacking exon 3 and 4 simultaneously; and isoform 5, in which the first 17 codons of exon 2 and 3 codons at the end of exon 5 are fused [109]. Isoforms 1, 2, and 4 are located only in the cytoplasm, whereas isoforms 3 and 5 are located both in the nucleus and in the cytoplasm. Each isoform has different binding affinities for downstream targets, differentially regulating the RAS signaling pathway. In addition, the different protein expression levels of each isoform are different; isoform 5 has shown almost 1000-fold lower expression than isoform 1. However, forced expression of isoform 5, which consists of 20 amino acids, has been reported to increase cell proliferation and transformation by activation of the NRAS target. The splicing isoform of A-Raf, called DA-Raf1, contains a Ras-binding domain, and it can bind to both Ras and M-Ras; however, it lacks a kinase domain and acts as a dominant-negative antagonist by interfering with the ERK pathway [110, 111].

*ERK1* exon 4 encodes a phosphorylation site for ERK1 kinase. SMNDC1, which is highly expressed in patients with pancreatic and ovarian cancers and associated with poor patient outcomes, has been reported to induce *ERK1* exon 4 inclusion [112]. Forced exclusion of exon 4 using antisense oligonucleotides has been shown to significantly reduce oncogenic ERK1, survival, and pro-apoptotic signaling and suppress target gene expression and tumor cell growth.

## Splicing factors that affect cancer progression

### RBM4

**Fig. 3:**
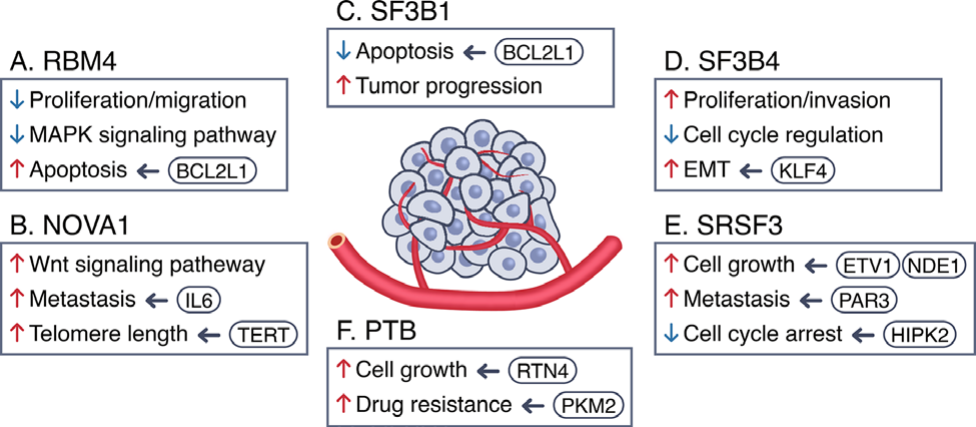
Key splicing factors that modulate cancer progression. Splicing factors play a crucial role in influencing cancer progression by modulating the process of alternative splicing. Several key splicing factors have been reported to significantly affect cancer progression. These factors include RBM4, NOVA, SF3B1, SF3B4, SRSF3 and PTB. Their specific roles involve regulating alternative splicing events in target gene pre-mRNAs that are associated with cancer progression. The intricate involvement of these splicing factors in alternative splicing contributes to the dynamic control of cellular processes related to cancer progression. Intracellular processes that are enhanced (red up arrows) or attenuated (blue down arrows) by these splicing factors are indicated, and the target genes involved are indicated

RNA binding motif protein 4 (RBM4), an RNA-binding protein that shuttles between the nucleus and cytoplasm, serves as a splicing factor that modulates alternative splicing by binding to the RNA recognition motifs (RRMs) GTAACG or CGGCGG, functioning as a general splicing inhibitor (Fig. 3A) [113, 114]. Following mRNA-sequencing analysis in RBM4-expressing H157 cells, alternative splicing events regulated by RBM4 were investigated using gene ontology. This analysis revealed that RBM4 target genes were associated with crucial cellular processes including cell proliferation, cell cycle regulation, apoptosis, migration, and tumorigenesis [115]. RBM4 demonstrated the ability to inhibit proliferation and migration in various cancer cell lines and effectively hindered cancer progression in tumor xenograft models [115–117]. Moreover, RBM4 influenced the utilization of the 5′ss of Bcl-x, an apoptosis regulator, inducing a shift from the anti-apoptotic isoform (Bcl-xL) to the pro-apoptotic isoform (Bcl-xS), thereby promoting apoptosis and impeding tumor progression [118, 119]. In addition, RBM4 downregulated the protein level of serine/arginine-rich splicing factor 1 (SRSF1), a splicing factor that functions as a proto-oncogene, and inhibited mTOR activation [120–122]. In gastric cancer cell lines, RBM4 has been shown to impede cancer progression by suppressing the expression of MAPK-dependent signaling pathway proteins [117]. Another study provided evidence that RBM4 exerted control over cell proliferation and mediated inflammatory responses by regulating the alternative splicing of transcription factors and co-activators [116]. Consistent with its role as a tumor suppressor, RBM4 expression has been found to be decreased in patients with NSCLC, breast cancer, pancreatic cancer, and gastric cancer [123]. In addition, patients with lung, breast, ovarian, and gastric cancers presenting higher expression of RBM4 exhibited higher survival rates, suggesting that RBM4 could serve be a target for human cancer treatment.

### NOVA1

Neuro-oncological ventral antigen 1 (NOVA1) is a well-known brain-specific splicing factor that plays a crucial role in alternative splicing regulation in neurons by binding to the YCAY sequence on target pre-mRNAs (Fig. 3B) [124–130]. In the context of gastric cancer, increased levels of miR-146b-5p in the surrounding tissues following gastrectomy have been associated with tumor recurrence and poor survival rates. Notably, miR-146b-5p inhibits NOVA1 expression in immune cells and stromal spindle cells within the remnant microenvironment [131]. Decreased NOVA1 levels in T cells within gastric cancer tissues are correlated with a decrease in FOXP3-positive regulatory T cells and are indicative of poor patient prognosis. These findings suggest that NOVA1 may serve as a potential biomarker for predicting the prognosis of gastric cancer patients and the presence of occult residual disease in remnant tissues post-gastrectomy.

Further studies by the same group found that attenuation of NOVA1 expression in T cells, tumor cells, and stromal spindle cells reduced FOXP3 + Treg density. In contrast, the infiltration of CD68 + macrophages and CD163 + M2 macrophages was increased, and NOVA1 expression was found to be associated with changes in immune cell composition [132]. Frequent NOVA1 inhibition was observed in the gastric cancer microenvironment, and decreased NOVA1 expression in tumor cells was strongly associated with tumor progression and poor prognosis. However, in contrast to these findings, NOVA1 mRNA has been reported to be significantly elevated in gastric cancer compared to that in non-tumor tissues, and high NOVA1 expression is associated with poorer prognosis [133]. In gastric cancer, miR-339 expression was downregulated, and mRNA and protein levels of NOVA1 were suppressed in miR-339-overexpressing cells. Overexpression of miR-339 inhibited gastric cancer cell proliferation, cell cycle progression, and invasion, and overexpression of NOVA1 impaired the inhibitory effect of miR-339 on gastric cancer cells. Another negative regulator of NOVA1 is miR-181b-5p, and its overexpression has been shown to suppress proliferation, migration, and invasion while promoting apoptosis in astrocytoma cells [134]. Similarly, downregulation of NOVA1 resulted in reduced cell growth, inhibited migration and invasion, and promoted apoptosis in U87 cells. Notably, patients with astrocytoma presenting high levels of NOVA1 expression exhibited worse survival outcomes compared with those with low NOVA1 expression levels.

Intratumoral Nova1 has been reported to be strongly correlated with hepatocellular carcinoma (HCC) poor survival and increased early recurrence [135]. HCC cell lines overexpressing Nova1 promoted cell proliferation, invasion, and migration. Nova1 plays a crucial role in promoting the inclusion of exon 9 in the inhibitory neurotransmitter receptor subunits GABA_A_Rγ2 [136–138]. Moreover, it interacts with the GABA_A_Rγ2 protein and demonstrates an inverse relationship with the expression levels of GABA_A_Rγ2 and GABA. Additionally, the upregulation of Nova1 expression promoted the growth of subcutaneous HCC in nude mice, indicating a potential oncogenic function for Nova1. Compared with normal lung tissue, NSCLC tissue showed significantly elevated NOVA1 expression, which correlated with indicators of poor differentiation, TNM stage, T stage, and lymph node metastasis [139]. Additionally, patients with NSCLC exhibiting high NOVA1 expression experienced shorter survival compared to those with low expression. NOVA1 facilitated the proliferative and invasive capacities of NSCLC cells by regulating the activation of the Wnt/β-catenin signaling pathway. Moreover, NOVA1 expression promoted the inclusion of exons 7 and 8 of human telomerase reverse transcriptase (*hTERT*), generating enzymatically active telomerase and influencing telomere length [140]. Notably, NOVA1 knockdown significantly reduced tumor growth in a xenograft model.

Additionally, NOVA1 has been associated with an unfavorable prognosis in patients with colorectal cancer (CRC) [141]. It upregulates MMP-2, MMP-7, and MMP-9 by regulating JAK2/STAT3 signaling through binding to and stabilizing *IL6* mRNA. Consequently, NOVA1 has been identified as a novel regulator that influences the proliferation and metastasis of CRC cells. NOVA1 has been reported to exhibit a tumor-suppressive effect depending on the specific environment; however, studies conducted till date collectively indicate that NOVA1 appears to have a dominant tumor-promoting effect affecting proliferation, invasion, migration, and telomerase activity.

### SF3B1

The SF3B complex regulates splicing of pre-mRNA by binding to the U2 snRNP and recognizing BPS [142]. The SF3B complex consists of seven proteins (SF3B1–7) with a molecular weight of 10 ~ 155 kDa. SF3B components, including SF3B1, have been implicated in cancer and various genetic disorders. Splicing factor 3B subunit 1 (SF3B1) is the largest protein within the SF3B complex, and it contains a HEAT [Huntingtin, elongation factor 3, subunit A of protein phosphatase 2 A, phosphatidylinositol 3-kinase (PI3K) target of rapamycin 1] domain consisting of 22 tandem repeats (Fig. 3C) [143]. The HEAT domain serves as the central region for RNA and protein binding in the SF3B complex. In human diseases, most *SF3B1* mutations are localized in the HEAT 4–12 region. *SF3B1* mutation has been detected in 30% of patients with myelodysplastic syndrome (MDS) and in 80% of patients presenting MDS subtype with ringed sideroblasts (RARS) [144–147]. Additionally, mutations have been detected in 20% of patients with MDS/myeloproliferative neoplasms and 15% of patients with chronic lymphocytic leukemia (CLL) [146, 148–150]. Furthermore, mutations in *SF3B1* have been reported at low frequencies in patients with acute myeloid leukemia, breast cancer, prolactinomas, uveal melanoma, leptomeningeal melanoma, blue nevus-like cutaneous melanoma, pancreatic ductal adenocarcinoma, and prostate cancer [151–159].

Although some reports have suggested no significant effect, SF3B1 mutations in patients with MDS have been associated with a favorable prognosis and long survival rates [146, 160–162]. However, in most diseases except MDS, but especially cancer, mutations in SF3B1 have been associated with poor prognosis and survival [163]. Furthermore, SF3B1 is overexpressed in glioblastoma, hepatocellular carcinoma, prostate cancer, and endometrial cancer [164–167]. Similar to its mutations, SF3B1 overexpression has been directly correlated with adverse patient prognosis, lower survival rates, and drug resistance. Dysregulation caused by SF3B1 mutations or changes in expression modulates oncogenic splicing variants such as BCL2L1-xL, KLF6-SV1, AR-v7 (androgen receptor variant 7), and In1-ghrelin [163–166, 168, 169]. The blockade or silencing of SF3B1 has been demonstrated to regulate the expression levels of essential components involved in mRNA homeostasis, including spliceosome, splicing factors, exon-junction complex (EJC) and SMG-1Upf1–eRF1–eRF3 (SURF) components, and NMD factors [164, 166]. Furthermore, it has been shown to modulate the AKT/mTOR/ß-catenin, JNK, PDK1, GSK3b, ERK, and AMPK signaling pathways [164–166]. Additionally, SF3B1 inhibition has been found to suppress proliferation, migration, apoptosis, and the formation of tumor spheres and colonies, as well as angiogenesis [164–166].

Capitalizing on the oncogenic properties of SF3B1 and its potential for suppressing tumor growth through blockade or silencing, several drugs have been developed to target SF3B1. These drugs include pladienolide B, spliceostatins, herboxidiene, sudemycins, and H3B-8800 [170–174]. Notably, H3B-800 entered phase I clinical trials in 2016, further affirming SF3B1 as a promising biomarker and a target for pharmacological treatment [175, 176].

### SF3B4

Splicing factor 3b subunit 4 (SF3B4) is a major subunit of the SF3B complex, consisting of two N-terminal RRMs and a C-terminal proline-rich (PR) domain (Fig. 3D) [177]. In addition to its role in pre-mRNA splicing, SF3B4 is implicated in cell signaling, transcription, and translation processes [178].

According to a TCGA analysis, only SF3B4 has shown high expression in the SF3B complex in cervical squamous cell carcinoma and endocervical adenocarcinoma [179]. SF3B4 has been reported to enhance the proliferation and invasion of cervical cancer cells, thereby promoting their malignant behavior. RNA-seq analysis conducted in SF3B4-knockdown HeLa cells revealed that differentially expressed genes (DEGs) were enriched in cellular processes such as regulation of cell proliferation, transcription, apoptotic process, and cell adhesion. Among the downstream targets of SF3B4, the gene exhibiting the most significant change in mRNA expression was sperm-associated antigen 5 (*SPAG5*). SF3B4 knockdown reduced SPAG5 expression by inducing the retention of *SPAG5* intron 21, subsequently causing premature termination of the transcript. SPAG5 is a mitotic spindle-binding protein involved in regulating mitosis [180]. It has been reported to promote the proliferation and progression of not only cervical cancer but also hepatocellular cancer and breast cancer by modulating the cell cycle [179, 181–184]. Notably, the presence of *SPAG5* intron retention transcripts correlated with extended survival times in patients. Collectively, these findings underscore the oncogenic role of SF3B4 in cervical cancer by virtue of its regulatory influence on *SPAG5* splicing.

SF3B4 has been reported to be up-regulated in hepatocellular carcinoma [185, 186]. High SF3B4 expression has been found to be associated with intrahepatic metastasis and poor prognosis [187]. Overexpression of SF3B4 has been shown to trigger the SF3b complex, which induced exon skipping of the tumor suppressor Kruppel-like factor 4 (*KLF4*), resulting in non-functional *KLF4* transcripts [188]. Consequently, cyclin-dependent kinase inhibitor 1B (*CDKN1B*; *p27Kip1*) became transcriptionally inactive, disrupting cell cycle regulation, while the activation of the Snail family transcriptional repressor 2 (*SNAI2*) gene promoted epithelial–mesenchymal transition (EMT). These events contributed to the malignant transformation and proliferation of liver cells. Furthermore, SF3B4 outperformed the existing diagnostic markers for hepatocellular carcinoma, namely glypican 3 (GPC3), glutamine synthetase (GS), and heat-shock protein 70 (HSP70) and demonstrated its potential as a reliable diagnostic marker for early-stage hepatocellular carcinoma.

Mutations in *SF3B4* mostly lead to reduced expression due to frameshifts, which cause acrofacial dysostosis, Nager syndrome, and Rodriguez syndrome [189, 190]. The *SF3B4* mutation affects the regulation of gene expression and abnormal splicing of crucial genes involved in skeletal development within growth plate chondrocytes [191, 192]. Consequently, this disruption ultimately manifests as defects in craniofacial and limb development, which are observed in acrofacial dysostosis.

SF3B4 has exhibited significant upregulation in ovarian cancer and a correlation with unfavorable patient prognosis [193]. The expression of SF3B4 has been found to be negatively regulated by miR-509-3p. RAD52, involved in DNA damage repair, assumes an oncogenic role in various tumors [194–196]. Loss of SF3B4 has been reported to reduce RAD52 expression by inducing retention of intron 8 of *RAD52* and generating premature termination codons. SF3B4 has been shown to act as an oncogene by modulating the alternative splicing of *RAD52*, thereby facilitating the proliferation, migration, and invasion of ovarian cancer cells. Additionally, SF3B4 has been reported to function as an oncogene in esophageal squamous cell carcinoma (ESCC) [197]. Conversely, in pancreatic cancer, the protein level of SF3B4 has shown a reduction compared with that in adjacent symptomatic tissue, and this diminished expression of SF3B4 has been found to facilitate the proliferation and migration of pancreatic cancer cells, indicating an inhibitory role for SF3B4 in pancreatic cancer [198]. These findings suggest that SF3B4 may have different biological functions, depending on the tumor type.

### SRSF3

The serine/arginine-rich splicing factor (SRSF) protein family comprises RNA-binding proteins that regulate various RNA biological processing such as mRNA transport and polyadenylation, as well as constitutive and alternative splicing [199–206]. Currently, 12 members (SRSF1–12) of the SRSF family have been identified in humans, with SRSF3 being the smallest member (Fig. 3E). SRSF3 has been observed to exhibit high expression levels in a wide range of tumors, including breast cancer, cervical cancer, colorectal cancer, gastric cancer, glioblastoma, head and neck squamous cell carcinoma, hepatocellular carcinoma, non-small cell lung cancer, oral squamous cell carcinoma, ovarian cancer, and retinoblastoma [203, 207–225].

SRSF3, a potential exonic splicing enhancer that is upregulated in glioblastoma, binds to the CA(G/C/A)CC(C/A) motif and alters more than 1,000 alternative splicing events [215]. Particularly, the knockout of SRSF3 leads to the exclusion of exon 7 in the *ETS* variant 1 (ETV1) gene and the replacement of the terminal exon 9 in the nudE neurodevelopment protein 1 (*NDE1*) gene with a mutually exclusive exon 9′. The ETV1 isoform with exon 7 inclusion and the NDE1 isoform with terminal exon 9 have been confirmed to be important for mitosis and cell proliferation of tumor cells, significantly increasing oncogenic activity. In addition, SRSF3 has been shown to inhibit PDCD4 protein expression by participating in alternative splicing, cytoplasmic export and translation of *PDCD4*, a tumor suppressor gene involved in antiproliferation, apoptosis, and antimetastasis [203, 226, 227].

MDM4 is an oncogene that suppresses the p53 tumor suppressor [228, 229]. SRSF3 is necessary for the inclusion of exon 6 (exon 7 in mice) in the human *MDM4* gene [230]. When this exon is excluded, it produces an unstable transcript containing a premature termination codon, which is subject to NMD. Consequently, this splicing event affects MDM4 protein levels. The in vitro and in vivo induction of *MDM4* exon 6 skipping using an antisense oligonucleotide have been reported to inhibit MDM4 protein abundance and melanoma growth and to increase sensitivity to MAPK-targeting therapeutics.

TAR DNA-binding protein (TDP43) is overexpressed in triple-negative breast cancer (TNBC) and is a major regulator of unique alternative splicing in TNBC [209]. SRSF3 interacts with these TDP43 to control specific splicing events, including that of *PAR3* and *NUMB*. SRSF3/TDP43 knockdown has been shown to inhibit cell proliferation by inducing *NUMB* exon 12 exclusion and inhibition of cell migration and invasion by inducing *PAR3* exon 12 inclusion. SRSF3 knockdown has been shown to induce G1 arrest and apoptosis by promoting downregulation of G1/S transition-related genes, BCL2 protein reduction, and homeodomain-interacting protein kinase-2 (*HIPK2*) exon 8 exclusion [214].

The oncogene Erb-B2 receptor tyrosine kinase 2 (ERBB2) is overexpressed in 20–30% of invasive breast cancer and is associated with poor prognosis [231]. In breast cancer cells, SRSF3, along with hnRNP H1, has been identified as a regulator responsible for controlling the production of distinct splice variants of *ERBB2* with different functionalities [232]. In particular, the knockdown of SRSF3 converted the oncogenic variant (exon 16 skipping) to a cell proliferation suppressive variant (premature stop codon generated by intron 15 inclusion). Additionally, SRSF3 binds to exon 18 of the interleukin enhancer-binding factor 3 (*ILF3*), leading to the production of an ILF3 isoform that facilitates cell growth [233]. Furthermore, SRSF3 has been demonstrated to be strongly related to the PI3K-AKT signaling pathway [201]. Cumulative evidence from various studies supports the role of SRSF3 in tumorigenesis, proliferation, and anti-apoptosis. Consequently, targeting the downregulation of SRSF3 holds potential as a therapeutic strategy for anticancer treatment.

### PTB

Polypyrimidine tract binding protein (PTB) shuttles between the nucleus and cytoplasm, and it is involved in various mRNA metabolic pathways, such as polyadenylation, mRNA stability, and initiation of translation, as well as regulation of pre-mRNA splicing (Fig. 3F) [234, 235]. PTB has a high affinity for binding to CU rich sequences including UCUU and CUCUCU [236, 237].

PTB is found to be overexpressed in epithelial ovarian tumors, glioma, and various cancer cell lines [238–240]. Knockdown of PTB has been shown to decrease cell proliferation, anchorage-independent growth, and invasiveness in these cell lines. Specifically, PTBP1 knockdown enhances the inclusion of exon 3 in reticulon 4 (*RTN4*) [241]. The presence of exon 3 in RTN4 isoforms is associated with reduced cell proliferation, suggesting that PTB-induced cell proliferation in glioma cells is partly mediated by *RTN4* splicing. Another study reported that in glioblastoma, PTBP1 recognizes an alternative 5′ss within ubiquitin specific peptidase 5 (*USP5*) exon 15, resulting in the generation of USP5 isoform 2 with a shorter exon length [242]. Enforced expression of USP5 isoform 1 through antisense targeting in glioblastoma cell lines has been demonstrated to inhibit cell growth and migration. These findings demonstrate that both the regulation of *RTN4* splicing by PTBP1 and the splicing of USP5 play important roles in gliomagenesis.

Keloid is a fibrotic skin disease characterized by excessive accumulation of extracellular matrix due to the proliferation of dermal fibroblasts [243, 244]. Its pathological features are similar to that of tumors, including tissue invasion and recurrence. PTB is overexpressed in keloid tissues and fibroblasts, and as in glioblastoma, alternative splicing changes in *RTN4* and *USP5* by PTB have been observed [245]. Furthermore, suppressing PTB has been shown to decrease the expression of fibronectin 1 (*FN1*) in transplanted keloid tissues and TGF-β1-treated keloid fibroblasts. FN1 is an important protein involved in cell adhesion, migration, and differentiation and is closely associated with cancer and fibrosis. Additionally, inhibition of PTB led to a reduction in excessive deposition of collagen type III alpha 1 chain (COL3A1), demonstrating that PTB siRNA promoted regression of keloid tissue in vivo by regulating both dermal cell proliferation and extracellular matrix accumulation.

PTB induces the exclusion of α-exon in fibroblast growth factor receptor 1 (*FGFR1*), which is associated with proliferation, and regulates the mutually exclusive splicing of exons IIIb and IIIc in fibroblast growth factor receptor 2 (*FGFR2*) [246, 247]. Moreover, PTB inhibits the inclusion of caspase 2 (*CASP2*) exon 9 and *FAS* exon 6, which are involved in apoptosis [248–250]. The binding of PTBP1 to pyruvate kinase M1/2 (*PKM*) intron 8 leads to the skipping of exon 9 in PKM, resulting in the generation of a PKM2 isoform that promotes the Warburg effect [251, 252]. This increased expression of PKM2 confers drug resistance in pancreatic ductal adenocarcinoma, suggesting that PKM2 and PTBP1 could serve as potential therapeutic targets to enhance the response to chemotherapy [253].

## Conclusions and future perspectives

After the development of RNA sequencing technology, comprehensive analyses of alternative splicing shed light on the remarkable diversity of mRNA variants in human genes. Recent studies have identified the functional importance of specific splicing isoforms and splicing factors in cancer progression, associated with regulation of cell numbers. Collectively, published and upcoming unpublished data uncover the effects of alternative splicing and provide the key to understanding the complexity of regulatory systems that determine cell fate. However, our understanding of the systemic regulation of alternative splicing by combinatorial activation or inhibition by hundreds of splicing factors and their fine-tuning control of the proteome is only at the initial stage. Moreover, short-lead bulk RNA sequencing, which has been the most common method for global splicing analysis, presents issues in precision when detecting alternative splicing patterns, especially for the small-size exons or transcripts with low expression levels. However, with the continuous development of alternative splicing analysis methods, such as long-read sequencing, and the application of bioinformatics techniques, such as deep learning, we expect that detailed cellular mechanisms can be profiled by analyzing not only gene expression levels but also alternative splicing patterns [254, 255].

In this review, we highlight the regulation of cancer cell proliferation through alternative splicing. Most studies have focused only on the expression level and PTMs of cell-number-regulating proteins. However, this approach is insufficient to fully explain the different functional outcomes of these genes depending on cancer types and stages, or individual patients with cancer. Recent studies have revealed that alternative splicing-mediated isoforms exhibit differential cellular functions in promoting cell growth. Therefore, alterations in alternative splicing within cancer cells can contribute to cell proliferation. Consequently, analyzing alternative splicing patterns in genes that regulate cell number may provide clues to identify the causes of abnormal cell growth and survival of cancer cells. Ultimately, exploration of global alternative splicing regulation with the role of individual splicing isoforms will suggest novel therapeutic splicing modulators or diagnostic markers for pathological splicing phenotypes and will provide a strategy to precisely predict therapeutic outcomes for precision medicine by revealing detailed information about tumor tissues.

## Data Availability

Not applicable.

## Abbreviations

CDS: Coding sequence
PTM: Post-translational modification
UTR: Untranslated region
snRNPs: Small nuclear ribonucleoproteins
snRNA: Small nuclear RNA
ss: Splice sites
BPS: Branch point site
PPT: Polypyrimidine track
CDK: Cyclin-dependent kinase
Rb: Retinoblastoma
CDKN1A: Cyclin dependent kinase inhibitor 1 A
CDKN2A: Cyclin dependent kinase inhibitor 2 A
CCND1: Cyclin D1
SAM68: SRC-associated in mitosis of 68 kDa
Ccnd2: Cyclin D2
ER: Endoplasmic reticulum
PCBP: Poly(C)-binding protein
E2F1: E2F transcription factor 1
E2F3: E2F transcription factor 3
NSCLC: Non-small cell lung cancer
TP53: Tumor protein P53
bHLH-LZ: Basic helix-loop-helix and leucine zipper
MAX: Myc-associated factor X
MXD3: MAX dimerization protein 3
TCGA: The Cancer Genome Atlas
Keap1: Kelch-like ECH-associated protein 1
Cul3: Cullin-3
Nrf2: Nuclear factor-erythroid factor 2-related factor 2
sMAF: Small musculoaponeurotic fibrosarcoma oncogene homologue
ARE: Antioxidant response element
HNSC: Head and neck squamous carcinoma
P: Promoter
tBHQ: Tert-butylhydroquinone
DGR domain: Double glycine-repeat domain
PIP2: Phosphatidylinositol (3,4)-bisphosphate
PIP3: Phosphatidylinositol (3,4,5)-trisphosphate
PH domain: Pleckstrin homology domain
PIK3R1: Phosphoinositide-3-kinase regulatory subunit 1
PR: Proline-rich motif
APDS: Activated phosphoinositide 3-kinase δ syndrome
MAPK: Mitogen-activated protein kinase
PDE6δ: δ-subunit of cGMP phosphodiesterase type 6
SRSF: Serine/arginine-rich splicing factor
NOVA1: Neuro-oncological ventral antigen 1
HCC: Hepatocellular carcinoma
hTERT: Human telomerase reverse transcriptase
CRC: Colorectal cancer
SF3B1: Splicing factor 3B subunit 1
PI3K: Phosphatidylinositol 3-kinase
MDS: Myelodysplastic syndrome
RARS: Ringed sideroblasts
CLL: Chronic lymphocytic leukemia
AR-v7: androgen receptor variant 7
EJC: Exon-junction complex
SURF: SMG-1Upf1–eRF1–eRF3
NMD: Nonsense-mediated mRNA decay
RRM: RNA recognition motif
SF3B4: Splicing factor 3b subunit 4
DEGs: Differentially expressed genes
SPAG5: Sperm-associated antigen 5
KLF4: Kruppel-like factor 4
CDKN1B: Cyclin-dependent kinase inhibitor 1B
SNAI2: Snail family transcriptional repressor 2
EMT: Epithelial–mesenchymal transition
GPC3: Glypican 3
GS: Glutamine synthetase
HSP70: Heat-shock protein 70
ESCC: Esophageal squamous cell carcinoma
ETV1: ETS variant 1
NDE1: nudE neurodevelopment protein 1
TDP43: TAR DNA-binding protein
TNBC: Triple-negative breast cancer
HIPK2: Homeodomain-interacting protein kinase-2
ERBB2: Erb-B2 receptor tyrosine kinase 2
ILF3: Interleukin enhancer-binding factor 3
PTB: Polypyrimidine tract binding protein
RTN4: Reticulon 4
USP5: ubiquitin specific peptidase 5
FN1: Fibronectin 1
COL3A1: Collagen type III alpha 1 chain
FGFR: Fibroblast growth factor receptor
CASP2: Caspase 2
PKM: Pyruvate kinase M1/2
